# Total extraperitoneal (TEP) versus laparoscopic transabdominal preperitoneal (TAPP) hernioplasty: systematic review and trial sequential analysis of randomized controlled trials

**DOI:** 10.1007/s10029-021-02407-7

**Published:** 2021-04-13

**Authors:** Alberto Aiolfi, Marta Cavalli, Simona Del Ferraro, Livia Manfredini, Francesca Lombardo, Gianluca Bonitta, Piero Giovanni Bruni, Valerio Panizzo, Giampiero Campanelli, Davide Bona

**Affiliations:** 1 Division of General Surgery, Department of Biomedical Science for Health, Istitituto Clinico Sant’Ambrogio, University of Milan, Milan, Italy; 2 Department of Surgery, Istituto Clinico Sant’Ambrogio, University of Insubria, Milan, Italy

**Keywords:** Inguinal hernia, Laparoscopic transabdominal preperitoneal repair (TAPP), Totally extraperitoneal repair (TEP), Recurrence, Chronic pain, Trial sequential analysis

## Abstract

**Purpose:**

To examine the updated evidence on safety, effectiveness, and outcomes of the totally extraperitoneal (TEP) versus the laparoscopic transabdominal preperitoneal (TAPP) repair and to explore the timely tendency variations favoring one treatment over another.

**Methods:**

Systematic review and trial sequential analysis (TSA) of randomized controlled trials (RCTs). MEDLINE, Scopus, Web of Science, Cochrane Central Library, and ClinicalTrials.gov were consulted. Risk Ratio (RR), weighted mean difference (WMD), and 95% confidence intervals (CI) were used as pooled effect size measures.

**Results:**

Fifteen RCTs were included (1359 patients). Of these, 702 (51.6%) underwent TAPP and 657 (48.4%) TEP repair. The age of the patients ranged from 18 to 92 years and 87.9% were males. The estimated pooled RR for hernia recurrence (RR = 0.83; 95% CI 0.35–1.96) and chronic pain (RR = 1.51; 95% CI 0.54–4.22) were similar for TEP vs. TAPP. The TSA shows a cumulative *z*-curve without crossing the monitoring boundaries line (*Z* = 1.96), thus supporting true negative results while the information size was calculated as adequate for both outcomes. No significant differences were found in term of early postoperative pain, operative time, wound-related complications, hospital length of stay, return to work/daily activities, and costs.

**Conclusions:**

TEP and TAPP repair seems comparable in terms of postoperative hernia recurrence and chronic pain. The cumulative evidence and information size are sufficient to provide a conclusive evidence on recurrence and chronic pain. Similar trials or meta-analyses seem unlikely to show diverse results and should be discouraged.

**Supplementary Information:**

The online version contains supplementary material available at 10.1007/s10029-021-02407-7.

## Introduction

Worldwide, more than 20 million patients suffer from inguinal hernia and undergo elective repair yearly [1, 2]. The Lichtenstein tension-free repair is the most commonly performed procedure with a low recurrence and complication rate [3]. Since the first description in the early ‘90s and because of the advent of innovative surgical platforms, the surgical technique evolved and the laparoscopic transabdominal preperitoneal (TAPP) repair and the totally extraperitoneal repair (TEP) emerged [4–10].

Compared to the Lichtenstein technique, minimally invasive approaches seem associated with a reduced risk of wound-related complication, early postoperative pain, return to work/activities, and chronic pain compared to the open approach [11]. The advantage of TEP repair is the nonviolation of the peritoneal cavity with the procedure totally performed in the preperitoneal space [12]. By contrast, TAPP repair requires peritoneal “violation” with the advantage of no technical/space constraints and the opportunity to provide a panoramic view of the myopectineal orifice with detection of unsuspected contralateral hernia when an adequate dissection of the preperitoneal space is achieved [13, 14]. Previous studies and meta-analysis described conflicting results for the direct comparison TEP vs. TAPP, while a robust indication of the best minimally invasive surgical option for the treatment of inguinal hernia remains unsettled [15–21].

The purpose of the present systematic review is to deeply assess the TEP vs. TAPP comparison for inguinal hernia repair in the setting of randomized controlled trials (RCT) and to perform a trial sequential analysis (TSA) to investigate if the required information size has been reached with conclusive evidence or oppositely if further trials and investigations are needed.

## Materials and methods

A systematic review was performed according to the guidelines from the preferred reporting items for systematic reviews (PRISMA) [22]. Institutional review board approval was not required. MEDLINE, Scopus, Web of Science, Cochrane Central Library, and ClinicalTrials.gov were used [23]. The last date of search was the November 30th, 2020. A combination of the following MeSH terms (Medical Subject Headings) were used: “Inguinal”, “Groin”, “Hernia”, “Herniorrhaphy”, “Mesh”, “Prosthetic material”, “Laparoscopic”, “Endoscopic”, “Transabdominal Preperitoneal” (TAPP), and “Totally Extraperitoneal” (TEP). Titles, abstracts, and references were evaluated. The PROSPERO study protocol was CRD42018091308.

### Inclusion and exclusion criteria

Inclusion criteria were: (a) RCT comparing surgical outcomes for elective inguinal hernia mesh repair for TAPP and TEP; (b) articles written in English; (c) when two or more papers were published by the same institution, study group, or used the same data-set, articles with the longest follow-up or the largest sample size; (d) in case of duplicate studies with accumulating numbers of patients, only the most complete reports were included for quantitative analysis. Exclusion criteria include: (a) observational non-RCT studies (b) non-English written; (c) non-clearly described methodology and technique; (d) single-arm studies; (e) studies with ≤ 15 patients per treatment arm.

### Data extraction

Extracted data include: author, year of publication, country, study design, number of patients, sex, age, body mass index (BMI), surgical approach, postoperative outcomes, quality of life, return to work/daily activities (days), cosmetic results, and costs ($). All data were independently computed by three investigators (SDF, LM, AA) and compared at the end of the reviewing process. A fourth authors (GC) reviewed the database and clarified discrepancies.

### Quality assessment

Three authors (AA, SDF, LM) assessed the methodologic quality of the selected trials using the Cochrane risk of bias tool [24]. This tool evaluates the following criteria: (1) method of randomization; (2) allocation concealment; (3) baseline comparability of study groups; and (4) blinding and completeness of follow-up. Trials were graded as having low (green circle), high (red circle), or unclear (yellow circle) risk of bias.

### Outcomes of interest

Primary outcomes include: chronic pain defined as as groin pain lasting for at least 3 months after the index procedure and hernia recurrence. Secondary outcomes include: early postoperative pain assessed with the Visual Analog Scale (VAS), wound-related complications (haematoma, seroma, and wound infection), operative time (minutes), hospital length of stay (HLOS), patients’-reported outcomes (quality of life and cosmetics), and costs ($). Outcomes were collected according to articles reporting. Haematoma was defined as any clinically diagnosed surgical site or scrotal hematoma. Seroma was defined clinically as a localized fluid filled sac that appeared on the operative site. Wound infection was defined as the presence of clinically diagnosed erythema, or purulent secretion or purulent secretion with fever. Hernia recurrence was defined clinically in all included RCT’s.

### Statistical analysis

The results of the systematic review were qualitatively summarized into frequentist study level random-effect meta-analysis of pooled risk ratio (RR) and standardized mean difference (SMD). An inverse-variance method and DerSimonian–Laird estimator for the variance of the true effect size (*τ*^2^) was performed [25]. Heterogeneity among studies was evaluated by the *I*^2^ index and Cochran’s *Q* test [26]. Statistical heterogeneity was considered low, moderate, and high for *I*^2^ values of 25, 50, and 75%, respectively, and significant when *p* < 0.10 [27, 28]. The Wald-type 95% confidence interval (CI) was computed for pooled measurements; otherwise, the 95% CI for the *I*^2^ index was calculated according to Higgins and Thompson [29]. The prediction interval for the treatment effect of a new study was calculated according to Borestein [26]. As the sample size was not the same in all studies, we performed a sensitivity analysis by excluding one study each time and rerunning the analysis to verify the robustness of the overall results. A two-sided *p* value was considered statistically significant when *p* < 0.05. All analyses and figures were carried out using the *R* software program, version 3.2.2 [30].

A TSA was performed to assess the possibility of type one error and to compute information size [31]. The Lan–DeMets approach was used to construct monitoring boundaries and to set adjusted thresholds for statistical significance. The information size was calculated at *α* = 0.05 and *β* = 0.2, with a risk ratio reduction (RRR) of 15% average survival and loss at follow-up of 1% [32]. The *z*-curve was constructed based on consecutive *z*-values, calculated using two-sided significant testing. The monitoring boundaries were constructed using conventional testing and by applying the O’Brien–Fleming *α*-spending function. The total number of observed patients in the cumulative meta-analysis was defined as the accrued information size (AIS). The TSA was performed using the Stata 14 software program [33].

## Results

### Systematic review

**Fig. 1:**
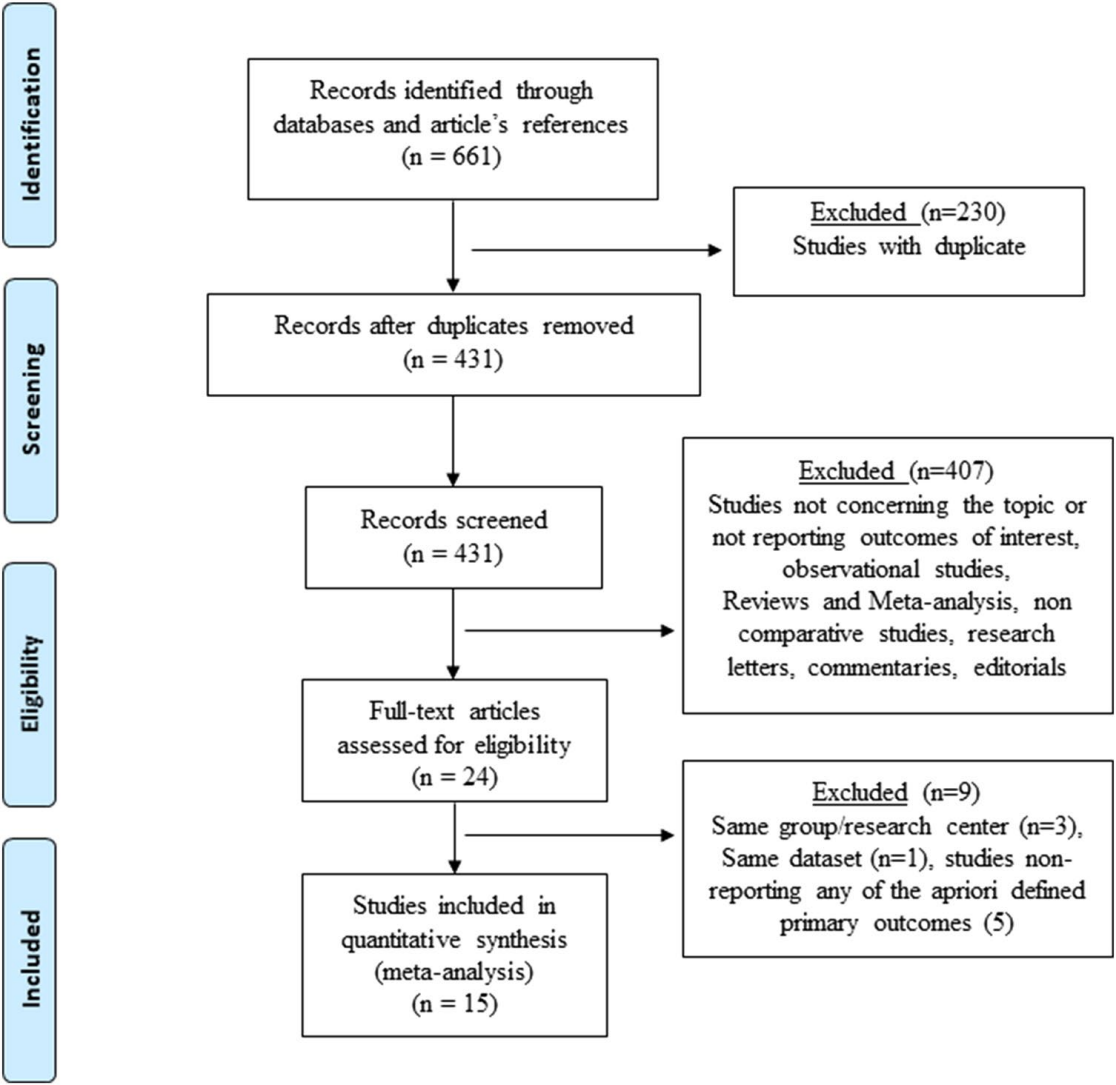
The Preferred Reporting Items for Systematic Reviews and meta-analysis checklist (PRISMA) diagram

The selection process flow chart is reported in Fig. 1. Initial search identified 661 publications. After removing duplicates, 431 titles and abstracts were reviewed. Further screening found 15 RCTs meeting the inclusion criteria. The included RCTs had issues regarding blinding taking into consideration that the application of blinding into surgical RCTs is challenging. The method of randomization was reported in 9 studies, while 13 RCTs described the operating surgeon’s proficiency. Details regarding the power analysis were specified in 4 studies (Supplementary Table 1). None of the studies received a low risk of bias on all assessed items. Because of the lack of patients and/or outcomes assessors blinding, all trials were graded as having high/unclear risk of performance and detection bias (Supplementary Figure 1).

Overall, 1359 patients were included in the analysis. Of these, 702 (51.6%) underwent TAPP and 657 (48.4%) TEP repair (Table 1). The sample size of the individual studies ranged from 40 to 314. The age of the patients ranged from 18 to 92 years and 87.9% were males. The American Society of Anesthesiologists (ASA) score and patients’ comorbidities were reported in six studies, while the BMI was reported in four trials. Overall, 86 (6.3%) underwent bilateral hernia repair while 60 patients (4.4%) were operated for recurrence. All trials reported the surgical technique, ten specified the type of hernia, and twelve reported the type of mesh and fixation techniques.

**Table 1:** Demographic and clinical characteristics of patients undergoing laparoscopic trans abdominal pre-peritoneal (TAPP) and totally extra peritoneal (TEP) repair

Author	Country	Surgical procedure	No. patients	Age (years)	Gender (male)	Type of hernia	Type of mesh	Follow-up (mos)
Schrenk et al. [34]	Australia	TAPP/TEP	28/24	39.1 ± 14.3 42.3 ± 11.9	24/22	Indirect: 19/18, Direct: 9/6	Polypropylene mesh (TAPP: SurgiPro®, Auto Suture®; TEP: SurgiPro®)	nr
Dedemadi et al. [35]	Greece	TAPP/TEP	24/26	65 (28–92)	nr	Nyhus class II 14/16, IIIA 7/8, IIIC 3/2	TAPP: nr	30 ± 1
							TEP: non absorbable mesh	
Gunal et al. [36]	Turkey	TAPP/TEP	39/40	25.7 ± 1 22.3 ± 0.6	nr	Nyhus class: I, II, IIIA, IIIB	Polypropilene mesh (6 × 12 cm)	nr
Butler et al. [37]	USA	TAPP/TEP	22/22	nr	nr	nr	Polypropylene mesh	1
Pokorny et al. [38]	Austria	TEP/TAPP	36/93	49 (19–73) 49 (21–78)	35/86	nr	Polypropylene mesh	36
Zhu et al. [39]	China	TAPP/TEP	20/20	62.3 ± 12 60.2 ± 9.7	19/20	nr	nr	nr
Hamza et al. [40]	Egypt	TAPP/TEP	25/25	36.7 ± 12 34.9 ± 13	25/25	Nyhus class I–III	nr	24
Krishna et al. [41]	India	TEP/TAPP	53/47	47.8 ± 16 51.3 ± 13.8	52/47	Indirect: 37/41, Direct: 26/18	Heavyweight polypropilene mesh (10 × 15 cm); preshaped 3Dmax polypropylene mesh	30
Gong et al. [42]	China	TAPP/TEP	50/52	56 ± 10 57 ± 9	50/52	Indirect: 35/37, Direct: 9/11, Both: 6/4	TAPP: polypropylene mesh (8.5 × 15 cm)	16 ± 8
							TEP: Bard® 3Dmax (8.5 × 13 cm)	
Mesci et al. [43]	Turkey	TAPP/TEP	25/25	48.2 48.4	nr	Indirect: 12/12 Direct: 8/7 Both: 5/7	nr	nr
Wang et al. [44]	China	TAPP/TEP	84/84	48.2 ± 13.2 52.1 ± 17.4	70/71	Indirect: 77/73, Direct: 6/8, Femoral: 1/3	TAPP: vypro II mesh (12 × 15 cm)	16 ± 7
							TEP: vypro II mesh (10 × 15 cm)	
Bansal et al. [45]	India	TEP/TAPP	160/154	50.7 ± 17.3 43.4 ± 16.4	nr	nr	Preshaped 3Dmax polypropylene mesh large size (Bard ®, 10.8 × 16 cm); Flat heavyweight polypropylene mesh (size 15 × 10 cm); Lightweight polypropylene mesh (Prolene soft, Ethicon®, 15 × 10 cm)	30 ± 14
Jeelani et al. [46]	India	TAPP/TEP	30/30	48.2 ± 13.3 46.7 ± 13	29/30	nr	Polypropilene mesh (10 × 15 cm)	24
Ciftci et al. [47]	Turkey	TEP/TAPP	30/31	44.4 ± 15.3 45.7 ± 11.1	26/26	Indirect: 20/20, Direct: 3/4, Both 7/7	Polypropilene mesh (15 × 8 cm)	3
Sharma et al. [48]	India	TAPP/TEP	30/30	49.4 49	59	Indirect: 33/28, Direct: 27/32	Polypropilene mesh (10 × 12 cm)	1

Data are reported as numbers, mean ± standard deviation, median (range)

*mos* months, *nr* not reported

All studies reported intraoperative complications; inadvertent hollow viscus injury was not reported, while inadvertent bleeding from right inferior epigastric artery was reported in 0.4% of patients (TAPP *n* = 1 and TEP *n* = 4). Conversion to minimally invasive approach to open repair was reported in 0.45% of patients for technical reasons (TAPP *n* = 2 and TEP *n* = 4), while conversion from TEP to TAPP was reported in 3 patients because preperitoneal adhesions (*n* = 2) and inadvertent peritoneal tear (*n* = 1). Postoperative follow-up duration ranged from 1 to 44 months. There were no mortalities.

### Meta-analysis and TSA: primary outcomes

**Fig. 2:**
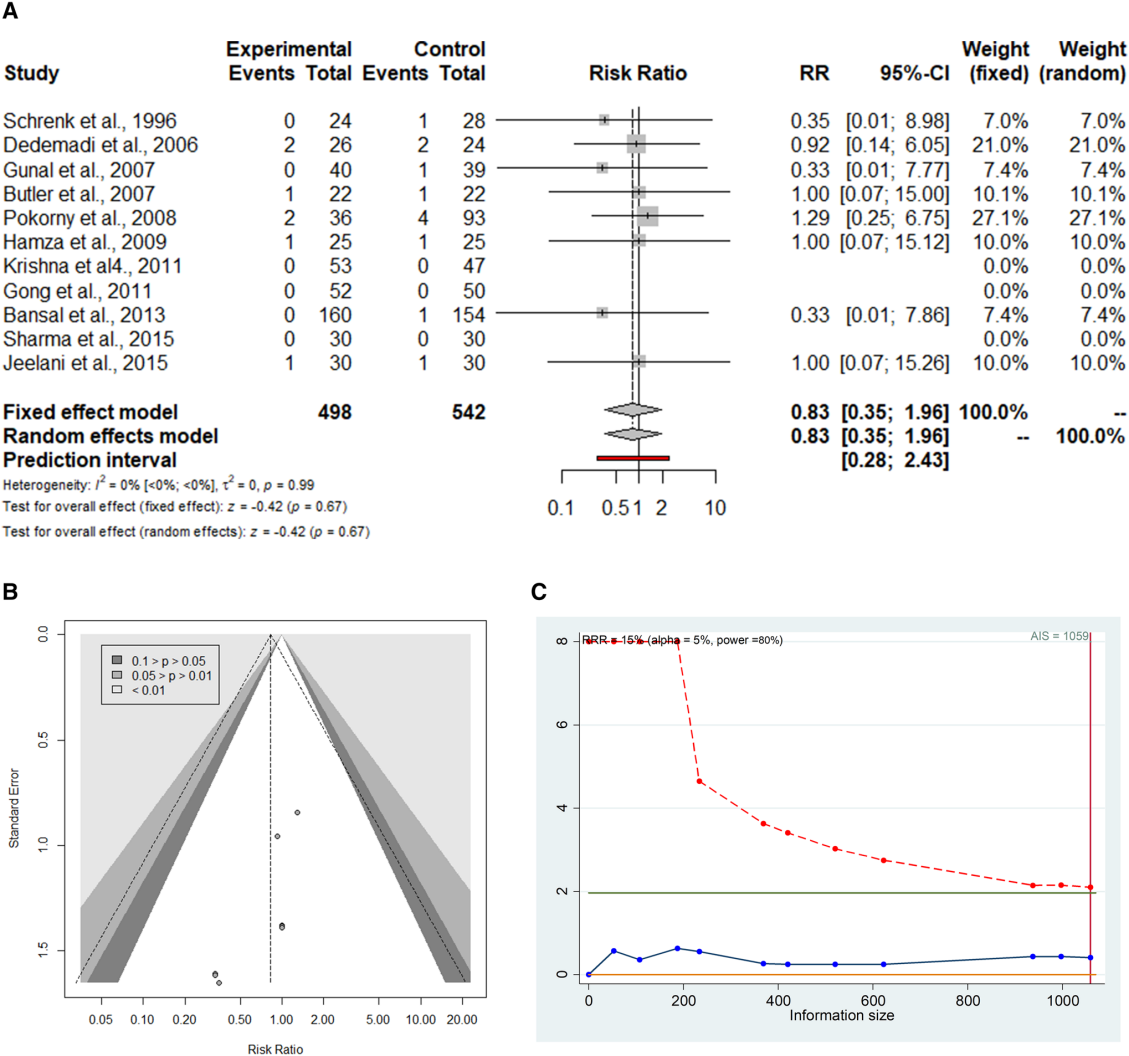
**a**–**c** Forrest plot (**a**), funnel plot (**b**) and trial sequential analysis (**c**) for postoperative hernia recurrence

Eleven studies (1040 patients) reported postoperative hernia recurrence with similar RR for TEP vs. TAPP (RR = 0.83; 95% CI 0.35–1.96) (Fig. 2a). The prediction lower and upper limits are 0.28 and 2.43, respectively. The heterogeneity is zero (*I*^2^ = 0.0%; 95% CI 0.0–0.0; *p* = 0.99) and *τ*^2^ = 0.0. Visual inspection of the Funnel plot does not show evidence of publication bias (Fig. 2b). The sensitivity analysis shows the robustness of these findings in terms of point estimation, relative confidence intervals, and heterogeneity. The sub-analysis including studies with > 12-month follow-up showed similar results (Table 2). The TSA, assuming an anticipated intervention effect of 15% RRR, shows a cumulative *z*-curve without crossing the monitoring boundaries curve (Fig. 2c). The required information size is reached and further trials are unlikely to demonstrate a statistically significant effect between the two techniques. This is a true negative result.

**Table 2:** League table

Categorical outcomes	RR (95% CI)	*I*^2^ (95% CI)	No. studies	No. patients
Hernia recurrence	0.83 (0.35–1.96)	0.0% (0.0–0.0%)	11	1040
Hernia recurrence (> 12-month follow-up)	0.95 (0.65–2.34)	0.0% (0.0–0.0%)	7	805
Chronic pain	1.51 (0.54–4.22)	0.0% (0.0–44%)	7	873
Chronic pain (> 12-month follow-up)	1.42 (0.63–3.84)	0.0% (0.0–26%)	6	821
Haematoma	1.19 (0.47–2.97)	0.0% (0.0–53%)	10	714
Seroma	1.24 (0.75–2.07)	0.0% (0.0–32%)	8	932
Wound infection	0.45 (0.17–1.17)	0.0% (0.0–0.0%)	9	916

Continuous outcomes	WMD (95% CI)	*I*^2^ (95% CI)	No. studies	No. patients
VAS < 12 h	− 0.42 (− 0.82; 0.12)	89% (81–93%)	9	923
VAS 24 h	− 0.35 (− 0.91; 0.22)	93% (89–96%)	9	891
VAS 48 h	− 0.06 (− 0.39; 0.27)	48% (0.0–81%)	5	285
VAS 1 week	− 0.41 (− 0.87; 0.18)	81% (56–92%)	4	508
VAS 1 month	− 0.29 (− 0.44; 0.14)	56% (35–77%)	4	516
VAS 3 months	− 0.38 (− 0.99; 0.22)	91% (77–97%)	2	414
Hospital length of stay (days)	0.22 (− 0.22; 0.66)	91% (85–94%)	11	1018
Operative time (minutes)	0.09 (− 0.41; 0.58)	95% (93–96%)	12	1188
Return to work (days)	− 0.03 (− 0.26; 0.21)	49% (0.0–77%)	8	691
Costs (US $)	0.46 (− 0.37; 1.29)	96% (93–98%)	4	684

Each row represents a specific outcome. Values in each column represent the relative effect for the comparison TEP vs, TAPP. Values are expressed as Risk Ratio (RR), weighted mean difference (WMD), and 95% confidence intervals (95% CI)

*I*^2^ Heterogeneity, *VAS* Visual Analog Scale

**Fig. 3:**
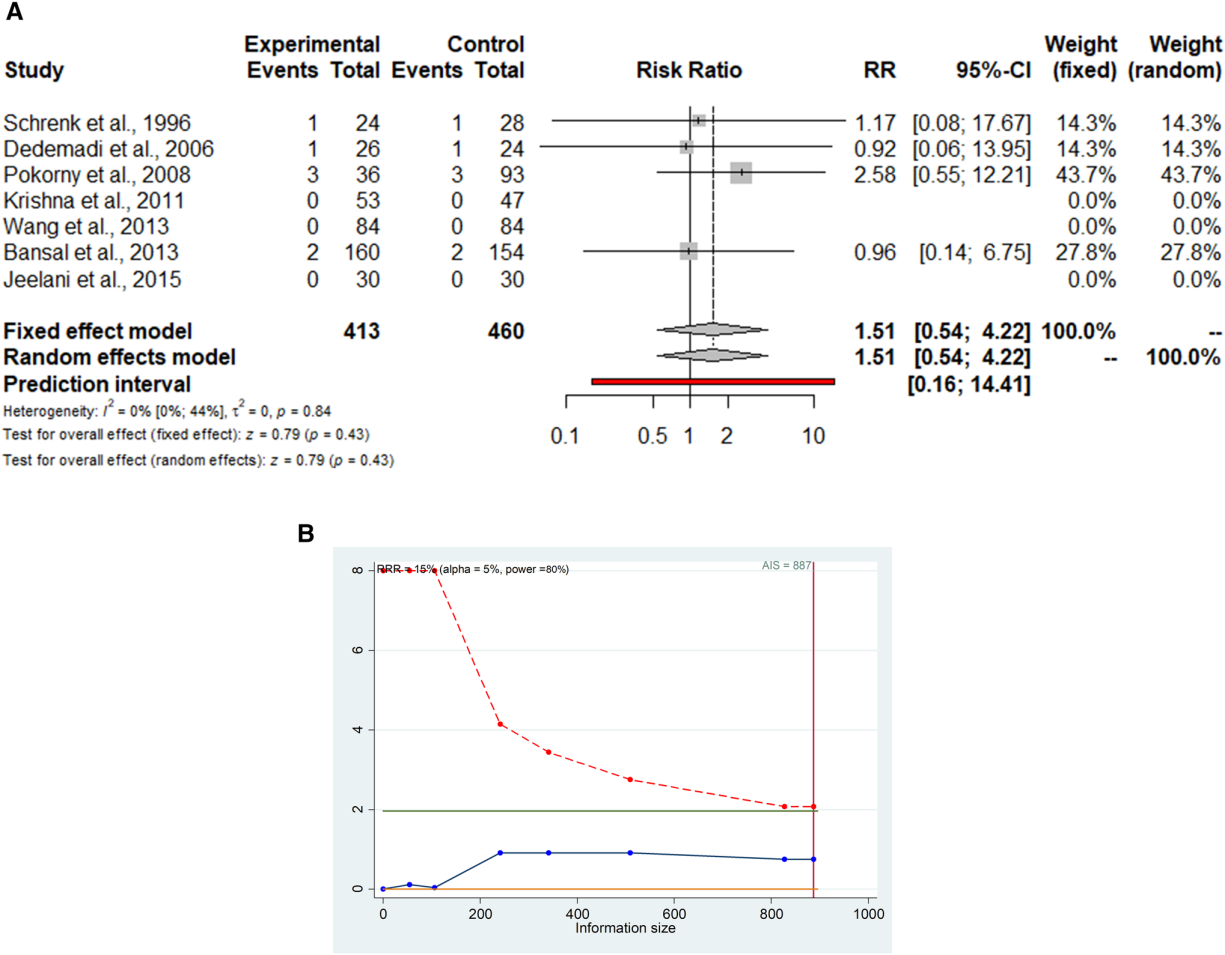
**a**–**b** Forrest plot (**a**) and trial sequential analysis (**b**) for postoperative chronic pain

Seven studies (873 patients) reported postoperative chronic pain with similar RR for TEP vs. TAPP (RR = 1.51; 95% CI 0.54–4.22) (Fig. 3a). The prediction lower and upper limits are 0.16 and 14.4, respectively. The heterogeneity is zero (*I*^2^ = 0.0%; 95% CI 0.0–44.0; *p* = 0.84) and *τ*^2^ = 0.0. The sensitivity analysis shows the robustness of these findings in terms of point estimation, relative confidence intervals, and heterogeneity. The sub-analysis including studies with > 12-month follow-up showed similar results (Table 2). The TSA, assuming an anticipated intervention effect of 15% RRR, shows a cumulative z-curve without crossing the monitoring boundaries curve (Fig. 3b). The required information size is reached and further trials are unlikely to demonstrate a statistically significant effect between the two techniques. This is a true negative result.

### Meta-analysis: secondary outcomes

The postoperative VAS score at < 12-h, 24 h, 48 h, 1 week, 1 month, and 3 months was similar for TEP vs. TAPP repair. Similarly, the analysis for wound-related complications showed equivalent postoperative hematoma, seroma, and wound infection RR. Operative time (WMD = 0.09; 95% CI − 0.41; 0.58) and hospital length of stay (WMD = 0.22; 95% CI − 0.22; 0.66) were similar between techniques. Eight studies (873 patients) reported return to work/daily activities with similar results for TEP vs. TAPP (WMD = − 0.03; 95% CI − 0.26; 0.21). The League table for all outcomes is reported in Table 2.

## Discussion

This meta-analysis shows that TEP and TAPP have comparable hernia recurrence and postoperative chronic pain. The trial sequential analysis shows that the information size is adequate, while future trials are unlikely to demonstrate a significant difference between the two techniques and should be avoided.

The recent European Hernia Society’s guidelines stated that Lichtenstein tension-free and minimally invasive techniques such as TAPP and TEP, performed by expert surgeons, are suggested as the best evidence-based options for inguinal hernia repair [1]. Approximately, 20% of patients with primary inguinal hernia underwent minimally invasive approach [49, 50]. Reasons of such a low percentage are probably related to higher direct costs and steep learning curve. However, a recent network analysis of RCT showed that both TEP and TAPP seem associated with reduced risk of postoperative pain and shorter return to work/daily activities compared to open tension-free repair [11].

Hernia recurrence after minimally invasive repair has been reported up to 2% for both TEP and TAPP repair [1–3]. Mesh type, size, and overlap, technique for mesh fixation (Self-gripping vs. sutured meshes vs. tacker vs. glue fixation), medial or lateral hernia sac, sliding hernia, operating time, type of anesthesia, participation in a register database, femoral hernia, adequate dissection and space creation, postoperative complications, and center/surgeon volume have been identified as risk factors [51–54]. The present analysis shows comparable results in terms of postoperative hernia recurrence RR. The global heterogeneity was zero (*I*^2^ = 0.0%) indicating a low degree of variability across studies, thus giving consistence to the result. This is similar to previously published studies and meta-analyses reporting similar postoperative hernia recurrence for TEP and TAPP repair [15–21]. Interestingly, no differences were found in the subgroup analysis including studies with > 12-month follow-up. Postoperative chronic pain after minimally invasive repair has been reported up to 3% [11]. The present quantitative analysis showed comparable RR for TEP vs. TAPP (RR = 1.51; 95% CI 0.54–4.22) (*I*^2^ = 0.0%) and no significant differences were found when considering studies with more than 12-month follow-up. This is in line with previously published studies that reported similar odds for chronic pain comparing TAPP and TEP repair [15–21]. So far, there is no conclusive evidence of differences in proportions for postoperative hernia recurrence and chronic pain for the TEP vs. TAPP comparison. Interestingly, the performed TSA suggested that the required sample was reached and the lack of statistical significance is a true negative results. Therefore, further trials or meta-analyses seem unlikely to demonstrate diverse results in term of postoperative hernia recurrence and chronic pain and should be avoided. These are key finding for the surgical community while research efforts should be focused on specific subgroups analysis (i.e., gender specifics, unilateral, bilateral, recurrence, high-risk patients, etc.), development of tailored strategies, patient-reported outcomes, and long-term follow-up (> 5 years).

A precise indication about the period of convalescence after inguinal hernia repair is lacking. Existing evidence suggest that patients should be encouraged to resume their activities as soon as they feel comfortable and return to work should be recommended if there is no excessive physical exertion and pain is controlled [52]. Significant variations exist because different confounders such as type of anesthesia, postoperative pain control, wound complications, preoperative patient expectations, motivation, culture, and administrative/insurance aspects may influence this aspect [55, 56]. In accordance with previously published studies, our results seem to further corroborate that both TEP and TAPP repair are associated with comparable return to work/daily activities. Early postoperative pain was comparable among treatments. This is in contrast with Chen et al. and Bansal et al. who reported a trend toward reduced postoperative analgesia requirement and reduced postoperative pain for TEP repair up to 1–3 months [21, 45]. The authors affirmed that the peritoneal incision and closure with continuous suture may be responsible for high pain scores for TAPP repair. However, not only the peritoneal incision is determinant of postoperative pain, but also several factors including patients’ subjective pain perception and expression, different protocols for anesthesia, postoperative analgesia, methods for mesh fixation (tacks vs. glue vs. self-gripping), mesh type, and weight (g/m^2^) have been shown to be additional causes of postoperative pain [56–59]. While our results seem to support equivalent postoperative pain, the related heterogeneity is moderate and caution is mandatory.

Surgeon experience, expertise, variation in technical skills, and hospital volume are key determinants for operative time while TAPP and TEP have been shown to be associated with a steep learning curve [60, 61]. The European Hernia Society stated that one hundred TAPP procedures are necessary to achieve comparable results with open mesh repair and that at least 50 cases are required to halve complication rates [1, 62]. Lau et al. affirmed that at least 80 TEP repair are required to complete the learning curve, while Aeberhard et al. reported a significant drop in surgery duration after one hundred procedures [63, 64]. In the present review, only three studies specified the operating surgeon proficiency [38, 44, 48], while the others reported that surgeries were performed by experienced surgeons. The appreciation of different anatomical landmarks in combination with the presence of tissue adherences or bleeding may increase the difficulties in the creation and maintenance of a proper preperitoneal working space in TEP repair, particularly in the early phase of the learning curve [12]. The accidental injury of the peritoneal layer, bleeding, and adhesions have been reported as possible causes of conversion to TAPP or open repair [48]. In the present study, conversion from TEP to open approach was reported in four patients because technical reasons, while three patients were converted to TAPP because of adhesions and inadvertent peritoneal tear. Some authors argued that TEP should be considered superior to TAPP repair because of the reduced risk of visceral injury. While this hypothesis is conceivable, in the present systematic review, none of the patients experienced inadvertent bowel or bladder injuries during TAPP. Notably, inadvertent injury of the inferior epigastric artery seems more frequent, while careful dissection should be performed to minimize this complication.

There are several limitations to the current analysis. First, although transitivity assumption was met with no evidence of statistically significant inconsistency, the accuracy of our results can be tempered by preoperative patients (men vs. women) and hernia characteristics (i.e., primary, recurrent, bilateral, etc.). Second, even though only RCTs were included in this review, the quality of evidence remained moderate, in part, due to the lack of patients, surgeons, and assessors blinding, limited power of some trials, different method for randomization and inclusion/exclusion criteria. Third, confounders related to surgeons’ experience and expertise, inclusion and exclusion criteria, learning curve, hospital volumes, operative technique, mesh type, fixation techniques, outcomes reporting, and follow-up may be additional confounders.

In conclusion, this systematic review and trial sequential analysis shows that TEP and TAPP repair seems comparable in terms of hernia recurrence and chronic pain. The cumulative evidence and information size are adequate to provide a conclusive evidence for both these outcomes. Similar trials or meta-analysis seem unlikely to show different results and should be discouraged.

## Supplementary Information

Below is the link to the electronic supplementary material.

Supplementary file1 Supplementary Figure 1. Risk of bias for Randomized Controlled Trials (RCT) was assessed with use of the Cochrane risk-of-bias tool. Green circle: Low risk of Bias. Red circle: High Risk of Bias. Yellow circle: Unclear Risk of Bias. (TIF 2809 KB)

Supplementary file2 (DOCX 18 KB)
