# Supplementary figures and images for: Total extraperitoneal (TEP) versus laparoscopic transabdominal preperitoneal (TAPP) hernioplasty: systematic review and trial sequential analysis of randomized controlled trials
Source: Hernia. 2021 Apr 13;25(5):1147–57. doi: 10.1007/s10029-021-02407-7 (PMC8514389; doi:10.1007/s10029-021-02407-7)

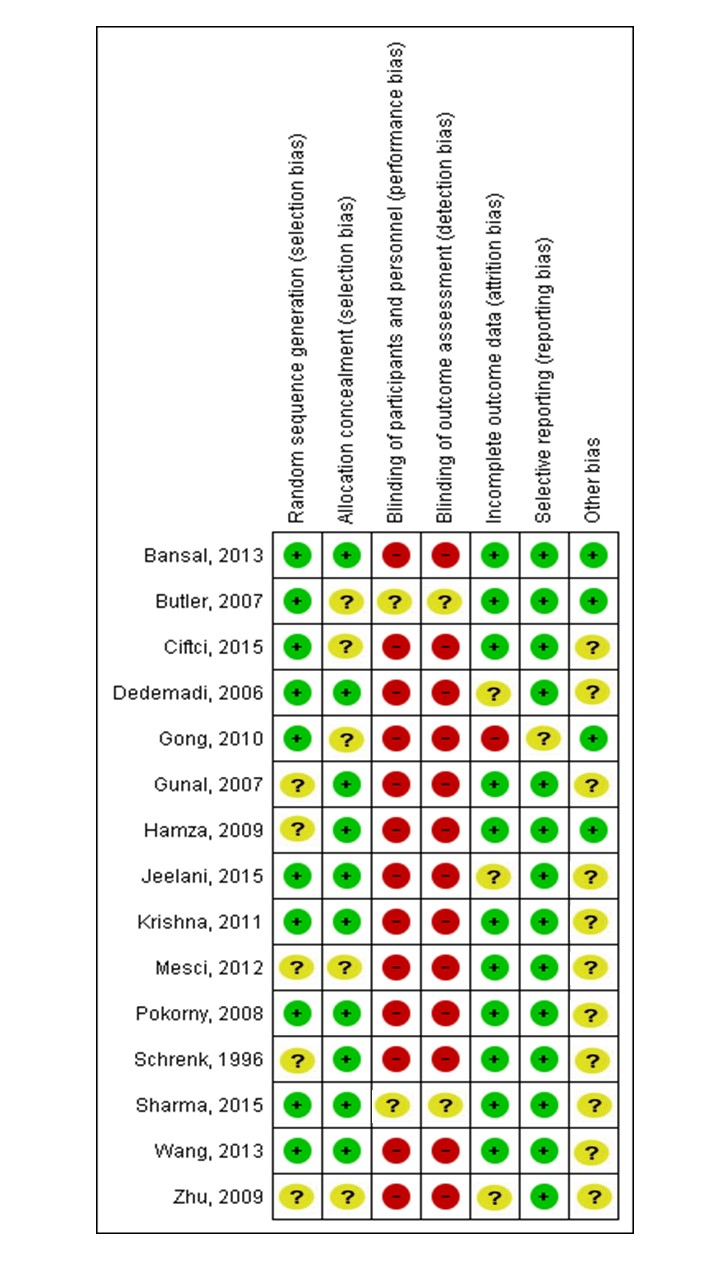

Supplement: Supplementary file 1 — Supplementary file1 Supplementary Figure 1. Risk of bias for Randomized Controlled Trials (RCT) was assessed with use of the Cochrane risk-of-bias tool. Green circle: Low risk of Bias. Red circle: High Risk of Bias. Yellow circle: Unclear Risk of Bias. (TIF 2809 KB) [file 10029_2021_2407_MOESM1_ESM.tif]
