# Supplementary material for: Total extraperitoneal (TEP) versus laparoscopic transabdominal preperitoneal (TAPP) hernioplasty: systematic review and trial sequential analysis of randomized controlled trials
Source: Hernia. 2021 Apr 13;25(5):1147–57. doi: 10.1007/s10029-021-02407-7 (PMC8514389; doi:10.1007/s10029-021-02407-7)
Supplement: Supplementary file 2 — Supplementary file2 (DOCX 18 KB) [file 10029_2021_2407_MOESM2_ESM.docx]

| **Author, year, country** | **Study design** | **Method of randomization** | **Surgeons' eligibility** | **Blinding** | **Power analysis** |
| --- | --- | --- | --- | --- | --- |
| Schrenk  et al, 1996 [33] | nr | enveloppe method | experienced surgeon | no | nr |
|  |  |  |  |  |  |
| Dedemadi  et al, 2006 [34] | nr | enveloppe method | experienced surgeon | no | Y |
|  |  |  |  |  |  |
| Gunal  et al, 2007 [35] | nr | nr | experienced surgeons | no | nr |
|  |  |  |  |  |  |
| Butler  et al, 2007 [36] | nr | nr | experienced surgeons | P: blind. S: blind | Y |
|  |  |  |  |  |  |
| Pokorny  et al, 2008 [37] | nr | stratified randomization | experienced surgeons (>30 TAPP repairs) | no | nr |
|  |  |  |  |  |  |
| Zhu  et al, 2009 [38] | nr | nr | nr | no | nr |
|  |  |  |  |  |  |
| Hamza  et al, 2009 [39] | nr | random number allocation | experienced surgeons | P: blind. S: blind. | nr |
|  |  |  |  |  |  |
| Krishna  et al, 2011 [40] | nr | Web-based randomization | experienced surgeons | no | nr |
|  |  |  |  |  |  |
| Gong  et al, 2011 [41] | nr | nr | experienced surgeons | no | Y |
|  |  |  |  |  |  |
| Mesci et al., 2012 [42] | nr | nr | experienced surgeon | no | nr |
|  |  |  |  |  |  |
| Wang  et al, 2013 [43] | nr | 1:1 randomization | experienced surgeons (>20 TAPP and TEP repair) | no | nr |
|  |  |  |  |  |  |
| Bansal  et al, 2013 [44] | nr | Randomization was performed using computer-  generated random numbers with sealed envelopes for  concealed allocation and block randomization in blocks of eight. | nr | no | Y |
|  |  |  |  |  |  |
| Jeelani  et al, 2015 [45] | nr | 1:1 randomization | experienced surgeon | no | nr |
|  |  |  |  |  |  |
| Ciftci et al., 2015 [46] | nr | nr | experienced surgeon | no | nr |
|  |  |  |  |  |  |
| Sharma et al., 2015 [47] | nr | computer generated random numbers | experienced surgeons (>100 TAPP/TEP repair) | P: blind. S: not blind. Stat: nr | nr |
|  |  |  |  |  |  |
|  |  |  |  |  |  |

**Supplementary Table 1**. Randomized Clinical Trials (RCTs) quality evaluation. TAPP: Laparoscopic Trans Abdominal Pre-Peritoneal, TEP Totally Extra Peritoneal, P patient, S surgeon, Y yes, nr not reported.
